# The complete mitochondrial genome sequence of *Siganus sutor* (Perciformes: Siganidae)

**DOI:** 10.1080/23802359.2017.1419101

**Published:** 2018-01-01

**Authors:** Wei Shi, Shixi Chen, Hui Yu

**Affiliations:** a College of Life Science, Foshan University, Foshan, Guangdong, China;; b CAS Key Laboratory of Tropical Marine Bio-resources and Ecology, South China Sea Institute of Oceanology Chinese Academy of Sciences, Guangzhou, China

**Keywords:** Perciformes, *Siganus sutor*, phylogenetic relationship

## Abstract

The complete mitochondrial genome of a marine fish *Siganus sutor* was completely sequenced by the high throughput sequencing method. This complete mitochondrial genome was 16,497 bp in length, consisted of 13 protein-coding genes, 22 tRNA genes, two rRNA genes and one large non-coding region. The gene arrangement of *S. sutor* is identical to those in typical fishes. Phylogenetic tree based on 13 protein-coding genes shows that Siganidae has a closer phylogenetic relationship to Luvaridae than to Ephippidae or Scatophagidae.

*Siganus sutor*, common name Shoemaker spinefoot, is a kind of coral reef fish (Grandcourt 2002). Long-distance movement of this fish have been reported (Kaunda-Arara and Rose 2004). They often occur among seagrasses to browse on ‘aufwuchs’ and form schools, and they occasionally eat poisonous fish (Gilbert 1993). The coloration of their body is highly variable; and influenced by substrate's colour and mood of the fish, upper colour is greenish to sandy brown, and the below is paler, with this colour patterns extending to the fins. In this study, we first reported the complete mitochondrial genome of *S. sutor* based on samples collected from Naozhou island in Zhanjiang, China (geographic coordinate: N 20°53′20.11″, E 112°28′46.2″), and analyzed its phylogenetic relationship with some other species from families Siganidae, Luvaridae, Scatophagidae, Ephippidae and Acanthuridae. The whole body specimen was preserved in ethanol and registered to the Marine Biodiversity Collection of South China Sea, Chinese Academy of Sciences, under the voucher number SCF20171022004.

The complete mitochondrial genome of *S. sutor* was 16,497 bp in length (GenBank accession No. MG677546), which included 13 protein-coding genes, two rRNA genes, 22 tRNA genes, one OL (origin of replication on the light-strand) and one D-Loop (control region). The OL was 50 bp in length, located in the cluster of five tRNA genes (*WANCY* region) between *tRNA*-*Asn* and *tRNA*-*Cys*. The D-loop was 831 bp in length, located between *tRNA*-*Pro* and *tRNA*-*Phe*. Gene arrangement of this genome was identical to that of those in typical fishes and most genes were encoded by the heavy strand (H-strand), except for *ND6* and eight tRNA genes (Inoue et al. 2001; Wang et al. 2017). Overall base composition values for the mitochondrial genome were 28.04%, 29.82%, 16.75%, and 25.39% for A, C, G, and T, respectively.

The phylogenetic relationships of *S. sutor* with 12 closely related species were analyzed in this study. The complete mitochondrial genes of these 13 species were available on GenBank. The Maximum Likelihood evolutionary tree (ML tree) was constructed by MEGA 7 (Kumar et al. 2016) based on 1st and 2nd codon sequences of 13 protein-coding genes.

In the ML phylogenetic tree, compared with other species, *S*. *sutor* and *S. fuscescens* have the closest phylogenetic relationship, and clade with *S. guttatus*, *S. puellus*, *S. unimaculatus* and *S. vulpinus* with a strong support. And these five species were all classified into family Siganidae of order Perciformes. *Luvarus imperialis* is a sister lineage to the formerly mentioned clade, *Ephippus orbis*, *Platax orbicularis* and *Platax teira* formed another clade, which was classified into family Ephippidae of order Perciformes. Another clade included *Scatophagus argus* and *Selenotoca multifasciata* with a strong support, which was classified into family Scatophagidae of order Perciformes (Figure 1). These results show that phylogenetic relationship between Siganidae and Luvaridae was closer than to Ephippidae and Scatophagidae.

**Figure 1.**
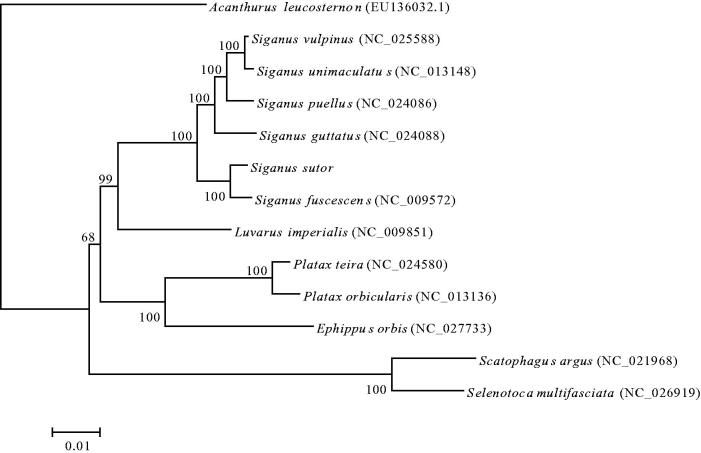
Maximum likelihood phylogenetic tree was constructed based on 1st and 2nd codon sequences of 13 protein-coding genes of 13 species.
